# Disentangling endogenous versus exogenous pattern formation in spatial ecology: a case study of the ant *Azteca sericeasur* in southern Mexico

**DOI:** 10.1098/rsos.160073

**Published:** 2016-05-25

**Authors:** Kevin Li, John H. Vandermeer, Ivette Perfecto

**Affiliations:** 1 School of Natural Resources and the Environment, University of Michigan, Dana Samuel Trask Building, 440 Church Street, Ann Arbor, MI 48109, USA; 2 Department of Ecology and Evolutionary Biology, University of Michigan, Kraus Natural Science Building, 830 North University, Ann Arbor, MI 48109, USA

**Keywords:** spatial distribution, clustering, point process models, biological control, agroecology

## Abstract

Spatial patterns in ecology can be described as reflective of environmental heterogeneity (exogenous), or emergent from dynamic relationships between interacting species (endogenous), but few empirical studies focus on the combination. The spatial distribution of the nests of *Azteca sericeasur*, a keystone tropical arboreal ant, is thought to form endogenous spatial patterns among the shade trees of a coffee plantation through self-regulating interactions with controlling agents (i.e. natural enemies). Using inhomogeneous point process models, we found evidence for both types of processes in the spatial distribution of *A. sericeasur*. Each year's nest distribution was determined mainly by a density-dependent relationship with the previous year's lagged nest density; but using a novel application of a Thomas cluster process to account for the effects of nest clustering, we found that nest distribution also correlated significantly with tree density in the later years of the study. This coincided with the initiation of agricultural intensification and tree felling on the coffee farm. The emergence of this significant exogenous effect, along with the changing character of the density-dependent effect of lagged nest density, provides clues to the mechanism behind a unique phenomenon observed in the plot, that of an increase in nest population despite resource limitation in nest sites. Our results have implications in coffee agroecological management, as this system provides important biocontrol ecosystem services. Further research is needed, however, to understand the effective scales at which these relationships occur.

## 1. Introduction

In the burgeoning literature on spatial ecology [1–4], a key problem remains understanding the processes whereby spatial pattern emerges. On the one hand, background physical–chemical–habitat pattern can create a template that the ecosystem of interest is forced to reflect; what might be called an exogenous mechanism of pattern formation [5–7]. On the other hand, a variety of modelling efforts leave little doubt that intrinsic biological forces can, in and of themselves, create spatial patterns under initially uniform background conditions [8–11]; perhaps the most elegant expression of which is the basic Turing mechanism [12], which has led to more general concepts of diffusive instability and the idea of ‘autonomous’, ‘endogenous’ or ‘self-organized’ pattern formation [11,13–15]. Apart from these extensive modelling studies, empirical evidence suggesting either endogenous or exogenous dominance in pattern structure is uncommon, perhaps because it is difficult to disentangle endogenous and exogenous contributions to spatial patterns [16]. Most of the empirical examples are associated with arid and semi-arid vegetation [17–19]. In these cases, the general conclusion is that endogenous forces, usually associated with competition for water, drive spatial pattern formation in arid and semi-arid systems (although exceptions exist [7]).

Less examined are the many cases of pattern formation in animal populations, most relevant to sessile species. Corals, for example, have been shown to develop spatial patterns based on various demographic features [20,21] as have mussel beds [22,23]. Ants are effectively sessile animals, because the colony is the effective organismal unit [24], and colonies typically abide in a nest that is fixed for a time in space. Several authors have noted that the classic ‘mosaic’ of spatial distribution of ant nests could result from basic endogenous variables [25,26] and, most importantly, noted the practical importance of understanding such spatial structure given that ants are keystone species in many tropical ecosystems [27].

We previously reported on the arboreal ant, *Azteca sericeasur* (formerly referred to as *Azteca instabilis*), likening its spatial pattern formation to diffusive instability (a Turing-like process) and leading to an intriguing example of ‘robust criticality’ [11,28]. *Azteca sericeasur* nests are engaged in the characteristic opposing forces of activation and repression of diffusive instability [11,29]: colony growth through nest ‘budding’ constitutes the activation process, whereby a new nest splits off an existing nest to colonize an empty tree; while attacks by natural enemies contribute to the repression component of the instability. These repression forces have been found to be density-dependent on ant nests [30–32].

Subsequently, we showed that the fact of a power law that led to the conclusion of robust criticality was dependent on the scale of cluster size examined [10]. The important issue of cluster scale remains understudied generally, especially at large scales. Is it the case that potential nesting sites 5 m removed from a current site would represent a nest within a defined cluster, but nesting sites 50 m away would not? What would be that critical clustering scale, and would it make a difference in terms of the observed spatial pattern? Might we be forced to conclude that pattern formation would be endogenous at one cluster scale but exogenous at a different scale? Such a separation of scales is often assumed just to make it possible to analyse endogenous and exogenous factors separately [33,34]. While our interrogations are cast in the context of one species of ant, however, we note that they are relevant to any sessile organism.

In this work, we take on these questions using a spatial dataset spanning 9 years in a permanent 45 hectare plot. Although our intent is mainly theoretical, seeking to provide empirical evidence in support or rejection of the hypothesis that the pattern is endogenous [10,11,35], the importance of this particular species with regard to provisioning the ecosystem service of pest control is clearly relevant. As a practical matter, this species' role as a biological control agent has been extensively reported [29,32,36–41], linking basic spatial ecological principles to the issue of ecosystem services [42].

Our approach first used spatial descriptive statistics to estimate the spatial relationship between nests. We then used a deductive ‘space as surrogate’ method [43], in which we developed specific spatial models that tested our *a priori* hypotheses of the ecological pattern-forming process. We fitted nest patterns from each year to point process models using two sets of spatial covariates representing hypothesized endogenous and exogenous contexts. Nest distributions in these models were alternately modelled under assumptions of spatial independence (a Poisson process) or dependence (a cluster process) between nests. The utility of adding the clustering process to our models was that it parametrized and fitted the pattern formation process within the spatial correlation analysis. Assuming that the clustering process adequately represented the biological reality, the fitted clustering parameters could then be used to make inferences about the spatial ecology of the system [44].

Our expectations were: (i) if endogenous processes played an important role in spatial pattern, nest distribution would have a significant density-dependent relationship over space and time, and (ii) if *A. sericeasur* nest distribution was influenced by density-independent exogenous processes, there would be significant correlations between nests and exogenous environmental heterogeneity, such as nest sites (i.e. trees) or abiotic variables. We compare the information explained by the fitted trends of the endogenous and exogenous covariates, interpret the fitted clustering parameters and the performance of the cluster process models, and discuss our results in the context of *A. sericeasur* population dynamics and management changes on the farm that reduced the total number of trees available for nesting.

## 2. Methods

### 2.1. Study plot

We conducted our study in a 45 hectare plot of an organic shade coffee plantation in the Soconusco region of Chiapas, Mexico, located approximately 92°20′29′′ W and 15°10′6′′ N at an elevation range between 900 and 1100 m. Like many areas of the tropics, this region experiences annual wet and dry seasons. Our data were collected annually in the wet season, between the months of May and July from 2004 to 2012. The farm was historically an organic, traditional polyculture coffee farm (see Moguel & Toledo [45] classifications), but began a shift to more intensified management (i.e. shade tree reduction) in 2007, which continued to the end of the focal period.

We mapped all trees on the plot that were greater than 10 cm in circumference at breast height (approx. 1.3 m) in annual transect surveys. Because *A. sericeasur* only nests within trees, tree coordinates also served as nest locations, the presence of which could be easily determined during the surveys, allowing us to map the complete spatial pattern of nests within the plot [11]. Between each year, trees were removed or added to the dataset when they died or were cut down, or had grown large enough to be included. The plot was georectified in 2012 using GPS (Trimble GeoXT) and a geographic information system [46].

### 2.2. Nest spatial clustering analysis

We used the pair correlation function (PCF) statistic [47,48], to quantify internest clustering within each year. The PCF test is related to the derivative of the Ripley's *K* test [49]. Both tests provide a population-wide summary of how individual points cluster with respect to other individuals as a function of an analysis scale *r*. The PCF differs in that it quantifies clustering only at a ‘ring’ of radius *r*, rather than a cumulative distance from 0 to *r* (i.e. a ‘disc’). The PCF can thus avoid confounding the effects of smaller-scale patterns with those at larger scales [48].

For each year, we performed a series of PCF tests over a 1–100 m range of scales. We compared the observed PCF statistic at each scale to the 95% confidence envelope of 1000 Monte Carlo random nest simulations. We generated these null nest patterns by randomly sampling without replacement from a pool of ‘available’ trees, which accounted for any underlying spatial patterns in the trees.

### 2.3. Modelling nest clustering and intensity with environmental variables

Point process models model point *intensity*, which is the probability of the occurrence of a point, given a location and a defined ‘process’, i.e. interaction rules with neighbouring points [50]. We used inhomogeneous point process models to fit nest patterns against exogenous and endogenous spatial variables that we hypothesized could have played a role in spatial distribution. We then considered the application of the Thomas cluster process [51], a member of the Neyman–Scott family of cluster point process models [50]. This is a still-developing area in point process modelling, which has seen increasing application in seed dispersal studies [52–55]. Our application of this cluster process to ant colony distribution is novel but appropriate, given the cluster-forming behaviour of *A. sericeasur*.

We fitted separate endogenous and exogenous models for each year of the study. Model variables were represented by spatial grids covering the entire plot. The endogenous models included the year-lagged ant nest density (*nest*) and its second-order term (*nest^2^*) to accommodate nonlinear density-dependent effects. The exogenous models fit tree density (*trees*), elevation (*elev*), *slope* and topographical wetness index (*wet*). Owing to the similar hill aspect across most of the plot, sun exposure was considered generally uniform throughout and was not included in the analysis.

Densities of trees and nests were calculated with a Gaussian kernel. We set the bandwidth (standard deviation) of the kernel at 20 m, which previous studies in this system have estimated as a reasonable radius defining clustering distance between nests and area of foraging influence [11,29]. This aligned with the observation from our PCF results that internest nest clustering tended to drop off beyond 40 m, as a bandwidth of 20 m placed 95% of the kernel density weighting within a 40 m radius of the kernel centre. However, it should be noted that while our decision was based on some experience and corroborated by our PCF results, the issue of optimal analysis scale and its implications in *A. sericeasur*'s spatial ecological interactions was not directly addressed in this study, though we do see it as a possible avenue of future research.

The abiotic environmental covariates were derived from a 20 m resolution digital elevation model using a GIS [46]. The slope grid was calculated as a function of the neighbouring values of each cell in the digital elevation model. Topographical wetness index, an approximation of water accumulation based on upstream catchment area and slope, was calculated by the function ln(*Ac*/*s*) for each cell, where *Ac* was the catchment area and *s* was slope [56,57].

To aid interpretation of the fitted model coefficients, these variable grids were scaled to have an approximately unit standard deviation across the plot and between all years of data, giving the coefficient estimates a uniform scale in terms of their variances across the study. Abiotic variable grids were normalized (mean = 0, s.d. = 1) to centre model predictions on average plot values.

We first modelled spatial variation in nest intensity by fitting an inhomogeneous Poisson process (IPP) model to nest points, using the endogenous or exogenous sets of spatial covariates. The IPP assumes no interaction between points, so any variation in intensity was attributed to spatial heterogeneity of the variables. We compared the relative information explained by each IPP model to a homogeneous Poisson process null, which assumed a uniform mean intensity of nests for the entire plot. We used the Akaike's information criterion (AIC) [58], which quantifies the deviance of a model penalized by its parameters, as the basis for comparison.

We added the assumption of Thomas clustering to the Poisson models, a two-generation process that first creates ‘mother’ points that are replaced by their ‘offspring’ clusters. The Thomas cluster process was an intuitive choice, because it uses an isotropic Gaussian shape to disperse offspring points around each cluster centre. This simulates the budding process of new colonies travelling outward from a mother nest and building daughter nests [29].

We fitted inhomogeneous Thomas cluster process (ITCP) models to the covariates. The fitting process assumed that the occurrence of mother points (*κ*) and the standard deviation of distance of offspring in a cluster from the mother (*σ*) were fixed for each year, whereas the number of points within each cluster (*µ*) varied with the environment and was determined from the IPP model. We used a two-step method [59] to model spatial variation in nest cluster intensity. The method uses the clustering shape of the empirical Ripley's *K* (weighted by predicted intensity from the IPP model) to optimize the theoretical Ripley's *K* function of the Thomas process, iteratively adjusting the *κ* and *σ* parameters to minimize the difference [60]. The fitted Thomas process parameters *κ* and *µ* are related to the IPP intensity by λ(*z*) = *κ* · *μ*(*z*), where λ(*z*) is the modelled IPP intensity at point location *z.* The coefficients that define *µ* are therefore the same as those fitted to the IPP model, although their uncertainties have been increased to reflect the effect of spatial clustering between points [61]. We used the statistical R package ‘spatstat’ to create all the point process models and perform the PCF analysis in §2.2 [62,63].

We obtained 95% confidence intervals for the *κ* and *σ* parameters of the fitted Thomas processes through parametric bootstrap of 999 simulations of the fitted models. The goodness-of-fit of the clustering patterns described by the ITCP models were assessed with the maximum absolute deviation (MAD) test [44], which determined whether a proposed model fit the observed spatial pattern by quantifying how often the model deviated from the actual pattern, according to a spatial statistic over a range of scales. We compared the MAD of the *L*-statistic, a variance-stabilized version of the Ripley's *K*, over 0–100 m for 999 simulations of all models, at a 95% acceptance level.

## 3. Results

### 3.1. General nest and shade tree trends

Ant nest populations had a significant increasing trend (slope = 46 nests year^−1^, *p *< 0.001, *R*^2^ = 0.85) over the study period. Tree population remained constant until tree thinning began in 2007. Afterwards, tree population decreased every year to the end of the study, at which point it was 60% of what it was in 2004. Exact nest and tree populations are given in the electronic supplementary material, S1.

### 3.2. Clustering analysis results

**Figure 1.**
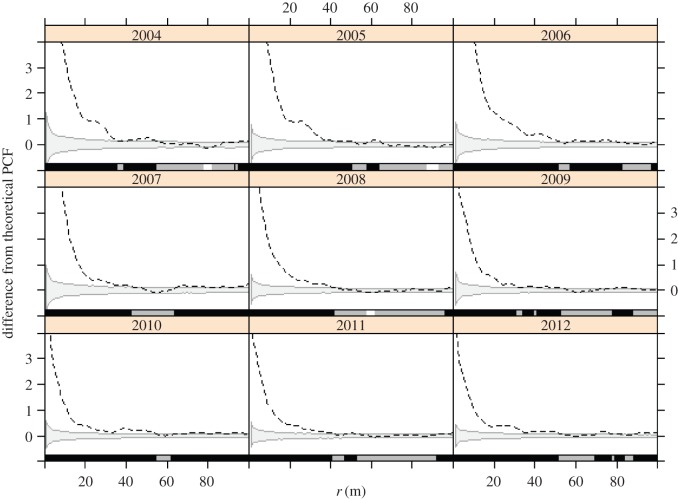
PCF statistic for annual nest distributions, plotted as the difference from the theoretical average expected value, represented by the dotted line. The grey area represents the 95% acceptance envelope of 1000 random nest allocations. The coloured bars along the *x*-axis indicate significant difference from the random patterns at that scale (black , clustered; white, dispersed; grey, not significant).

PCF results are reported in figure 1. The amount of clustering in the graph is quantified as the difference from a theoretical random (Poisson) distribution defined by the mean point intensity over the entire plot. The shaded regions represent the 95% acceptance envelopes for the null hypothesis of random nest patterns, as determined by the Monte Carlo reassignment of nests to trees.

The shape of the function suggested significant clustering for scales ranging from 1 m to 30–60 m with the maximum depending on the year, although smaller peaks at larger scales could represent ‘echoes’ of the main scale of interaction [44]. Disregarding these peaks, the most important clustering appears to have occurred within the range of 0–40 m for every year. Qualitatively, the shape of the curves changed as well. In the last 3 years, the observed PCF statistic dropped steeply relative to the theoretical value with increasing *r*, whereas in the first 3 years, there was more area under the curve at distances less than *r* = 20 m. This indicates that in later years, nest patterns became closer to random at scales under 20 m, possibly reflecting an underlying change in tree spatial structure owing to thinning. Nests remained clustered within this structure, however, as the existing patterns were still significantly different from random reallocations within each year's tree distribution. Maps of nest locations are provided in the electronic supplementary material, S2.

### 3.3. Nest cluster modelling results

Table 1 reports the fitted variable coefficients and significance at *α* = 0.05 for the endogenous and exogenous IPP and ITCP models. Because the Thomas process was fitted to the IPP trend, the coefficients for both models are the same, but the uncertainty of the ITCP increases to account for clustering.

**Table 1.** Fitted spatial covariate coefficients of the inhomogeneous Poisson process (IPP) and inhomogeneous Thomas cluster process (ITCP) models. (Each year (column) of a table represents a separate set of IPP and ITCP models. Coefficients are the same between models but have different confidence intervals. Significant coefficients are in italics and indicated by dagger or asterisk for significance in the IPP or ITCP model, respectively. The *p*-values are reported in the parentheses below each coefficient, with the IPP *p-*value listed on top.)

	2005	2006	2007	2008	2009	2010	2011	2012
exogenous model								
*int.*	−7.11	−7.34	−7.06	−7.01	−7.42	−7.51	−7.64	−7.56
*elev*	0.04	*0*.*24*^†^	0.11	0.02	0.04	*0*.*14*^†^	0.02	0.05
	(0.52)	(0.0007)	(0.13)	(0.71)	(0.49)	(0.007)	(0.66)	(0.37)
	(0.79)	(0.16)	(0.48)	(0.87)	(0.73)	(0.24)	(0.83)	(0.65)
*slope*	−*0*.*15*^†^	−0.09	−0.07	0.03	−0.09	−*0*.*13*^†^	−0.07	−*0*.*15*^†^
	(0.04)	(0.24)	(0.33)	(0.64)	(0.14)	(0.02)	(0.25)	(0.008)
	(0.37)	(0.61)	(0.64)	(0.83)	(0.44)	(0.28)	(0.54)	(0.16)
*wet*	0.00	0.04	0.00	−*0*.*11*^†^	−0.07	0.02	0.04	−0.02
	(0.96)	(0.50)	(0.97)	(0.03)	(0.17)	(0.69)	(0.38)	(0.58)
	(0.98)	(0.75)	(0.99)	(0.22)	(0.42)	(0.83)	(0.62)	(0.74)
*trees*	0.02	0.04	−0.02	0.08	*0*.*22*^†^	*0*.*26*^†*^	*0*.*34*^†*^	*0*.*36*^†*^
	(0.71)	(0.40)	(0.69)	(0.08)	(0.0003)	(0.0000)	(0.0000)	(0.0000)
	(0.87)	(0.73)	(0.85)	(0.43)	(0.08)	(0.02)	(0.01)	(0.004)
endogenous model								
*int.*	−8.86	−9.17	−8.82	−7.87	−8.35	−8.05	−8.27	−8.30
*nest*	*2*.*09*^†*^	*1*.*69*^†*^	*1*.*52*^†*^	*1*.*16*^†*^	*1*.*18*^†*^	*1*.*18*^†*^	*1*.*07*^†*^	*1*.*26*^†*^
	(0.0000)	(0.0000)	(0.0000)	(0.0000)	(0.0000)	(0.0000)	(0.0000)	(0.0000)
	(0.0000)	(0.0002)	(0.0000)	(0.0000)	(0.0000)	(0.0000)	(0.0000)	(0.0000)
*nest^2^*	−*0*.*26*^†*^	−*0*.*16*^†^	−*0*.*15*^†*^	−*0*.*09*^†^	−*0*.*09*^†*^	−*0*.*10*^†*^	−*0*.*08*^†*^	−*0*.*11*^†*^
	(0.0000)	(0.0000)	(0.0000)	(0.0000)	(0.0000)	(0.0000)	(0.0000)	(0.0000)
	(0.0009)	(0.07)	(0.01)	(0.22)	(0.0000)	(0.03)	(0.0003)	(0.0001)

When assuming no nest clustering in the endogenous models (IPP), elevation was significant in 2006 and 2010 with a positive coefficient; slope was significant in 2005, 2010 and 2012 with a negative coefficient; and wetness was significant in 2008 with a negative coefficient. The ITCP exogenous models did not assign significant coefficients to any abiotic variables. Tree density was significant with a positive coefficient in IPP and ITCP models in 2010, 2011 and 2012, and significant for just the IPP model in 2009.

**Figure 2.**
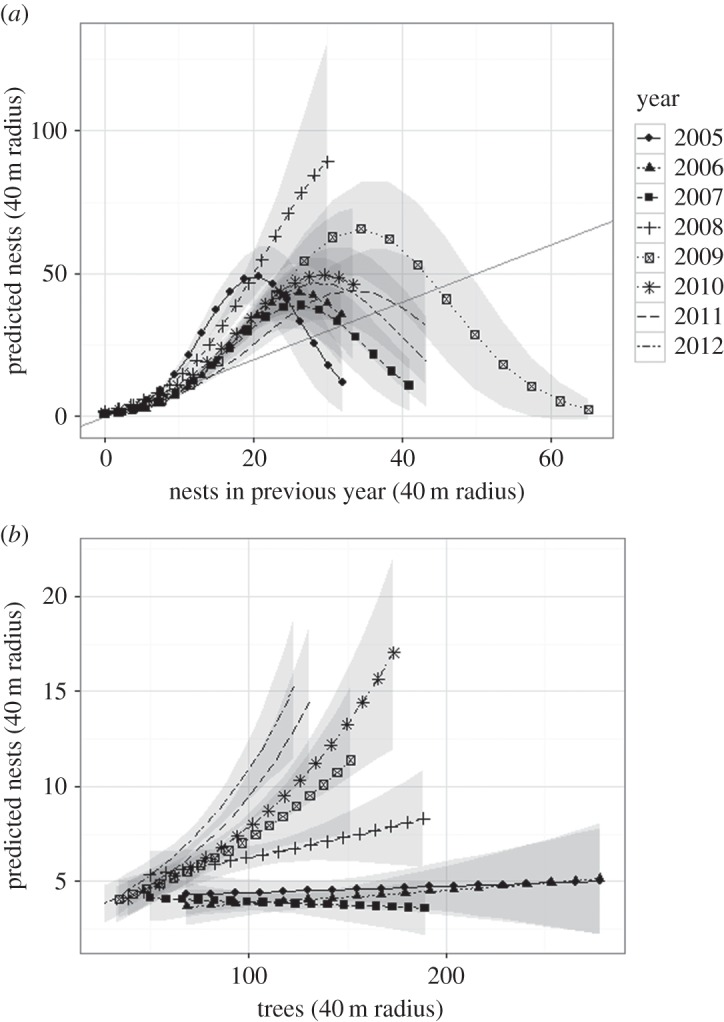
Predicted nest density and 95% confidence intervals for each year from (*a*) the endogenous model (varying lagged nest density), and (*b*) the exogenous model (varying tree density). Predicted density was derived from the intensity value predicted by the models. All spatial covariates were held at the plot mean except for the *x*-axis variable. The plotted range of each year reflects the actual range for that year.

We plotted the predicted nest density of the exogenous model against the number of trees in a 40 m radius (figure 2*b*). These graphs show that in the first 3 years of our models, the probability of finding a nest at a given point was unrelated to the number of trees in its 40 m radius. After 2007, nest occurrence became more likely with an increasing number of surrounding trees. This corresponds with the initiation of the tree felling campaign on the farm that decreased the tree population and maximum tree density found on the plot. This could reflect an increasing dependence of nest density on tree availability, although this relationship may be indirect, as the projected nest density remained much lower than the predicting tree density.

In the endogenous models, lagged nest density was a significant positive predictor for both the IPP and ITCP models. The second-order term was significant for every year in the IPP models, but was not significant in 2006 and 2008 in the ITCP models. The negative coefficient of the second-order variable suggests a nonlinear relationship between lagged nest density and predicted density, which we plot in figure 2*a*. Figure 2*a* demonstrates a hump-shaped curve of nest occurrence predicted by the number of nests in a 40 m radius in the previous year. The diagonal line in figure 2*a* represents the ‘break-even’ line, where the predicted nest density would equal the previous year's nest density. The shape of these curves support the idea that nest growth and survival are nonlinear and density-dependent, as the predicted nest occurrence is greater than the diagonal line at lower lagged nest densities, but switches over (i.e. crosses the ‘break-even’ line) at higher lagged densities. This threshold increased from around 30 in the 3 years before tree thinning (2005–2007), to a significantly greater peak above 40 in 2009 (the 2008 model only predicted density increase, as the 2007 nest density levels were not sufficient to cross the projected threshold), and returned to a lower level around 35 in 2010–2012. At very low levels of previous nest density, the predicted nest density also fell below the previous year's density every year, suggesting an Allee effect for very isolated nests.

The AIC of the endogenous and exogenous IPP models and a homogeneous Poisson process null are reported in table 2. In the first 5 years, exogenous models added relatively little information to their respective null models, compared with the last 3 years' models, which improved AIC by at least 27. Endogenous models decreased (improved) in AIC from the null model by much more—approximately 500 for each model.

**Table 2.** Comparison of AIC values of the exogenous and endogenous inhomogeneous Poisson process models, and a homogeneous Poisson process null model. (Datasets are shared within years, so AIC values are comparable by column. Relatively lower AIC values indicate that more information is explained by that model.)

	2005	2006	2007	2008	2009	2010	2011	2012
null	6031	5637	5240	7856	7763	9289	8729	9560
exogenous	6031	5624	5244	7851	7746	9237	8702	9510
endogenous	5432	5004	4782	7355	7277	8808	8276	9053

The fitted parameters of the ITCP models are reported with their bootstrapped 95% confidence intervals in table 3. Qualitatively, the endogenous models fit smaller values of *σ* (standard deviations of dispersal distance) and larger values of *κ* (cluster centre intensities) than the exogenous models. In some years, these differences were significant. Within each set of models, fitted *σ*-values were not significantly different across the years of the study, but *κ*-values had an increasing trend that paralleled the increase in nest population in the plot and were significantly different between some years of the first and second half of the study period.

**Table 3.** Inhomogeneous Thomas cluster model results and 95% confidence intervals. (Fitted parameter κ is the intensity of the cluster centre Poisson process and *σ* is the standard deviation of the Gaussian spread of each cluster. Maximum absolute deviation (MAD) measures the goodness of fit of the proposed model, based on the transformed Ripley's *K, L*(*r*) = sqrt(*K*(*r*)/pi). A MAD value below 0.05 is outside the 95% confidence interval that the model process describes the same pattern as the actual distribution.)

	exogenous model			endogenous model		
	σ	*κ* × 10^4^	MAD	*σ*	*κ* × 10^4^	MAD
2005	7.4 (5.7, 8.8)	1.9 (1.4, 2.9)	0.55	2.6 (1.7, 5.5)	7.3 (2.9, 14.6)	0.05
2006	8.6 (6.6, 10.4)	1.6 (1.1, 2.5)	0.70	3.1 (2.2, 6.5)	2.7 (1.1, 4.9)	0.02
2007	7.0 (5.3, 8.4)	2.1 (1.5, 3.3)	0.51	3.6 (2.6, 6.2)	2.4 (1.1, 4.0)	0.02
2008	8.2 (6.3, 9.6)	3.2 (2.4, 4.9)	0.61	6.1 (4.7, 9.1)	4.8 (2.7, 7.1)	0.06
2009	8.4 (6.5, 10.2)	3.8 (2.9, 5.9)	0.40	2.8 (1.8, 5.5)	20.1 (9.3, 39.1)	0.02
2010	11.1 (8.4, 13.8)	3.5 (2.4, 5.7)	0.28	4.0 (3.1, 5.6)	9.5 (6.1, 14.2)	0.04
2011	9.7 (7.4, 11.8)	4.3 (3.0, 6.8)	0.50	3.7 (2.5, 6.2)	16.8 (9.2, 31.2)	0.01
2012	8.7 (6.7, 10.5)	5.4 (3.9, 8.3)	0.30	6.1 (4.3, 10.0)	9.6 (5.1, 17.5)	0.03

Table 3 also reports the results of goodness-of-fit tests for the clustering models. Low goodness-of-fit *p-*values for the MAD test indicated that the endogenous models were significantly different from the actual original nest distributions, and thus not appropriate representations of clustering. From plotting simulated *L-*test results of both models against the actual pattern (electronic supplementary material, S3), it was evident that the endogenous ITCP models over-predicted clustering, whereas exogenous models encompassed actual clustering patterns. This may have been because the Thomas process in the endogenous models conflicted with the lagged nest density covariates, which already accounted for aspects of the nest clustering process. On the other hand, the Thomas process of the exogenous models was able to account for all aspects of multigenerational nest distribution, and could thus simulate clustering accurately. For this reason, we consider only the clustering parameters of the exogenous ITCP models in our discussion.

## 4. Discussion

Our results demonstrate how Poisson and cluster point process approaches, previously limited to vegetation studies in ecology, can provide insights into the spatial distribution and clustering of other sessile organisms such as ants. We determined a relevant scale of clustering for *A. sericeasur* nests, although with limitations. PCF results suggested that significant clustering occurred below 40 m, whereas the exogenous Thomas clustering parameters suggested an average *σ* of 8.6 m, placing 95% of nests within 17.2 m of cluster centres. These results are remarkably similar, assuming the maximum significant PCF clustering scale describes nests on the farthest ends of a cluster, which gives an effective cluster radius of about 20 m. However, both these methods assumed a simplified interpretation of nest dispersal, ignoring multigenerational processes or approximating their patterns as only offspring nests, though actual distributions were a combination of offspring and survivors. Adding multigenerational cluster processes with more than two generations [55] is a possible direction of future modelling efforts.

Although endogenous and exogenous processes are often considered in isolation for ecological study, in reality, species patterns probably reflect a combination of both [64]. Regardless, consideration should be given to environmental heterogeneity outside the pattern-forming process of interest when interpreting spatial patterns [65]. The uniformly and lushly treed environment of this organic shade coffee farm may have originally formed an effectively homogeneous environment for the arboreal *A. sericeasur* and its interacting species [11,29,66]; and within this environment, the clustered ant nest distribution suggested an autonomous diffusive-instability pattern-formation process [10,11,28]. However, our study reveals the changing nature of the interactions in this system and the emerging significance of environmental context.

Two consistent patterns emerged from our point process modelling: exogenous models indicated that tree density became an increasingly important positive predictor of nest density, whereas endogenous models suggested a changing density-dependent relationship between nests. However, the discrepancy in AIC between the two models indicated that endogenous effects still explained more of the present patterns. Decreasing nest density predictions from high previous density supported the findings of prior work in this system, perhaps reflecting the repressing effects of parasitoid phorid flies that attack at higher rates in large nest clusters and areas with more ant activity [31], or similar responses by natural enemies of *A. sericeasur*'s scale insect mutualist *Coccus viridis*, such as the hyperparasitic fungus *Lecanicillium lecanii* [28] or the coccidophagous beetle *Azya orbigera* [35]. Our models also suggested an Allee effect at very low nest densities, although it is unclear why small, isolated clusters would experience higher mortality. This result opens a line of questioning that has not yet been considered by the rich body of empirical work done in the system.

A previous paper [10] points out a counterintuitive aspect of this system's dynamics, wherein the reduction of a presumed limiting resource, trees for nesting sites, is accompanied by an increase in the ant nest population. That study demonstrated through cellular automata modelling that escape from a more dispersal-limited natural enemy presents a plausible hypothetical mechanism for nest increase. From the point of view of natural enemies, tree reduction would decrease nest density at a local scale and increase distances between nests, potentially hindering movement and counter-balancing a response to increases in plot-scale nest density. This could have led to a change in the dominant controlling natural enemy, proposed originally to be phorid flies [11], as that species lost its effectiveness in exploiting the nest population.

This study found empirical evidence that the negative control on nest densities did indeed change in character, whatever its species identity. The threshold nest density above which negative control became dominant in the subsequent year (i.e. the intersection of the curve with the ‘break-even’ line in figure 2*a*) shifted significantly. This threshold peaked after the start of the tree thinning programme and returned close to initial levels in later years, a trend that could be indicative of a transition in the controlling regime. However, we did not see evidence of increased nest dispersal that would support the dispersal-limited natural enemy hypothesis in our Thomas cluster models. The fitted clustering parameters suggested that the dispersal distance of the nests within nest clusters did not change significantly, even in later years when management on the farm changed. Rather, only the intensity of cluster centres increased. The hypothesis was also contradicted by the significant positive relationship between nest density and tree density in later years, as we would have expected more nests with lower tree density, because this would have been a condition for greater internest distances.

When considering these trends, and as is often the case in ecological spatial analysis and spatial correlation studies, scale is of critical importance [67,68]. We attempted to make an informed choice in the scale of our correlations by basing spatial variables on previous experience in the system, a choice that was supported by the PCF results. However, the assumption that this scale best represents how a colony interacts with conspecifics and the environment remains untested explicitly. Future modelling efforts should employ a scale-explicit framework [69] to investigate whether the components of the ecological pattern-forming process operate at significantly different scales or have changed differently, while also considering multiple levels of clustering [55]. The ‘appropriate’ scale of spatial analysis could be identified through minimizing either the residual spatial autocorrelation of these relationships [67] or the pattern discrepancy in spatial point process modelling [52]. Clarifying the scale of these relationships would be an important step in understanding how *A. sericeasur* interacts with its ecosystem and environment, and give more clues to the shifting density-dependent mechanism acting on the nest population in this plot.

The importance of understanding these patterns beyond a theoretical interest should be emphasized. *Azteca sericeasur* is a well-established keystone species in a system that provides biological control of critical agricultural pests of coffee [27,38,70,71]. Furthermore, the spatial distribution of *A. sericeasur* affects the distribution of other important ecosystem service providers, such as the natural enemies of *C. viridis* [29,35], among which *L. lecanii* is also a hyperparasite and spatial driver of the economically important coffee rust fungus *Hemileia vastatrix* [28,32,72]. Understanding the dynamics behind spatial distribution is crucial for effective utilization of ecosystem services [42], enabling agroecosystem managers in diversified farming systems to predict and respond to ecological disturbances, promoting resilience in the face of a changing climate and other environmental pressures.

## Supplementary Material

“Electronic supplementary material” is a pdf file containing additional figures and tables that provide the reader with more details about our results.

## Data accessibility

The associated data of this study are openly accessible on the Dryad online data repository at: http://dx.doi.org/10.5061/dryad.487mp. Statistical software and packages used in this study were the R software environment, available at: https://www.r-project.org/ and the spatstat spatial analysis package, available at: http://spatstat.github.io/.
