# Supplementary material for: Disentangling endogenous versus exogenous pattern formation in spatial ecology: a case study of the ant Azteca sericeasur in southern Mexico
Source: R Soc Open Sci. 2016 May 25;3(5):160073. doi: 10.1098/rsos.160073 (PMC4892448; doi:10.1098/rsos.160073)
Supplement: “Electronic supplementary material” is a pdf file containing additional figures and tables that provide the reader with more details about our results. [file rsos160073supp1.pdf]

Electronic supplementary material 1: Nest and tree population, 2004-2012. Tree felling began in the plot in 2007.

Year	*Total trees*	*nests*
2004	10595	282
2005	10595	378
2006	10595	350
2007	8834	322
2008	8560	513
2009	6425	506
2010	7294	624
2011	6145	581
2012	5876	644

Electronic supplementary material 2: Distribution of ant nest points in the plot for years 2004-2012. Tree-felling began in 2007, eventually reducing the number of potential host sites for ant nests by one-third.

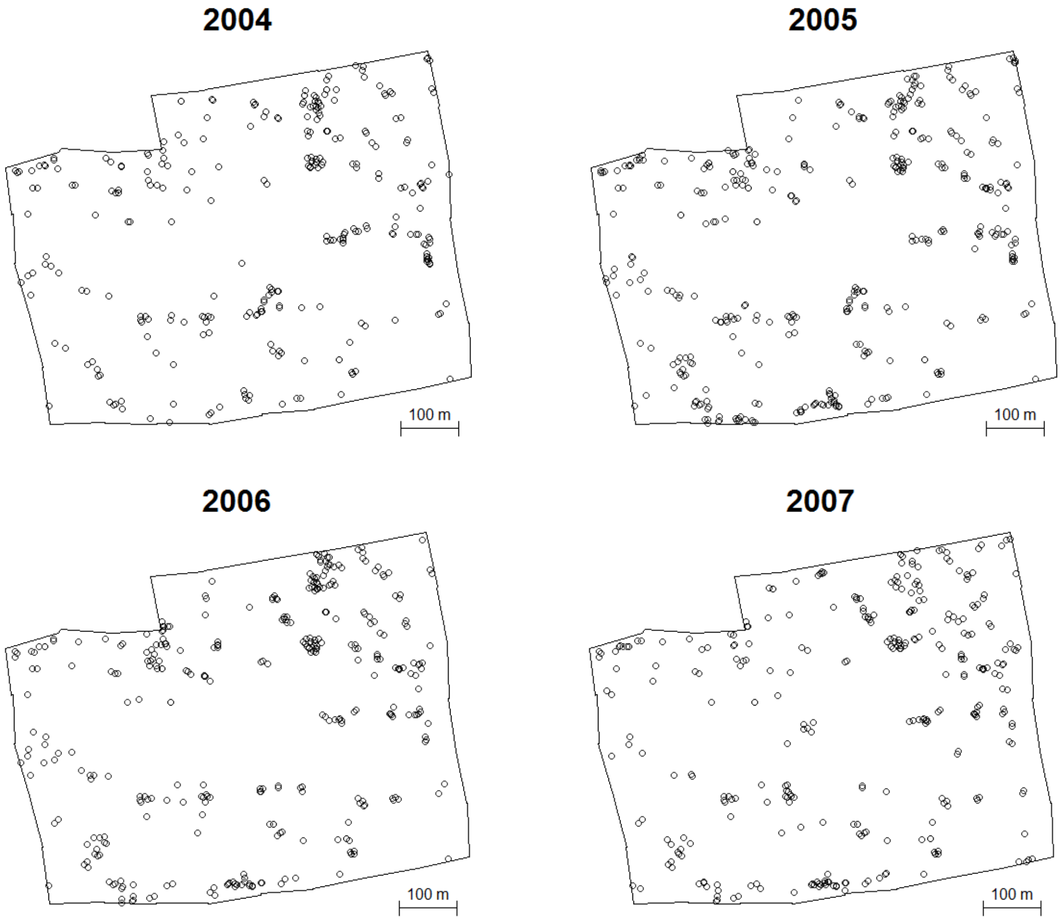

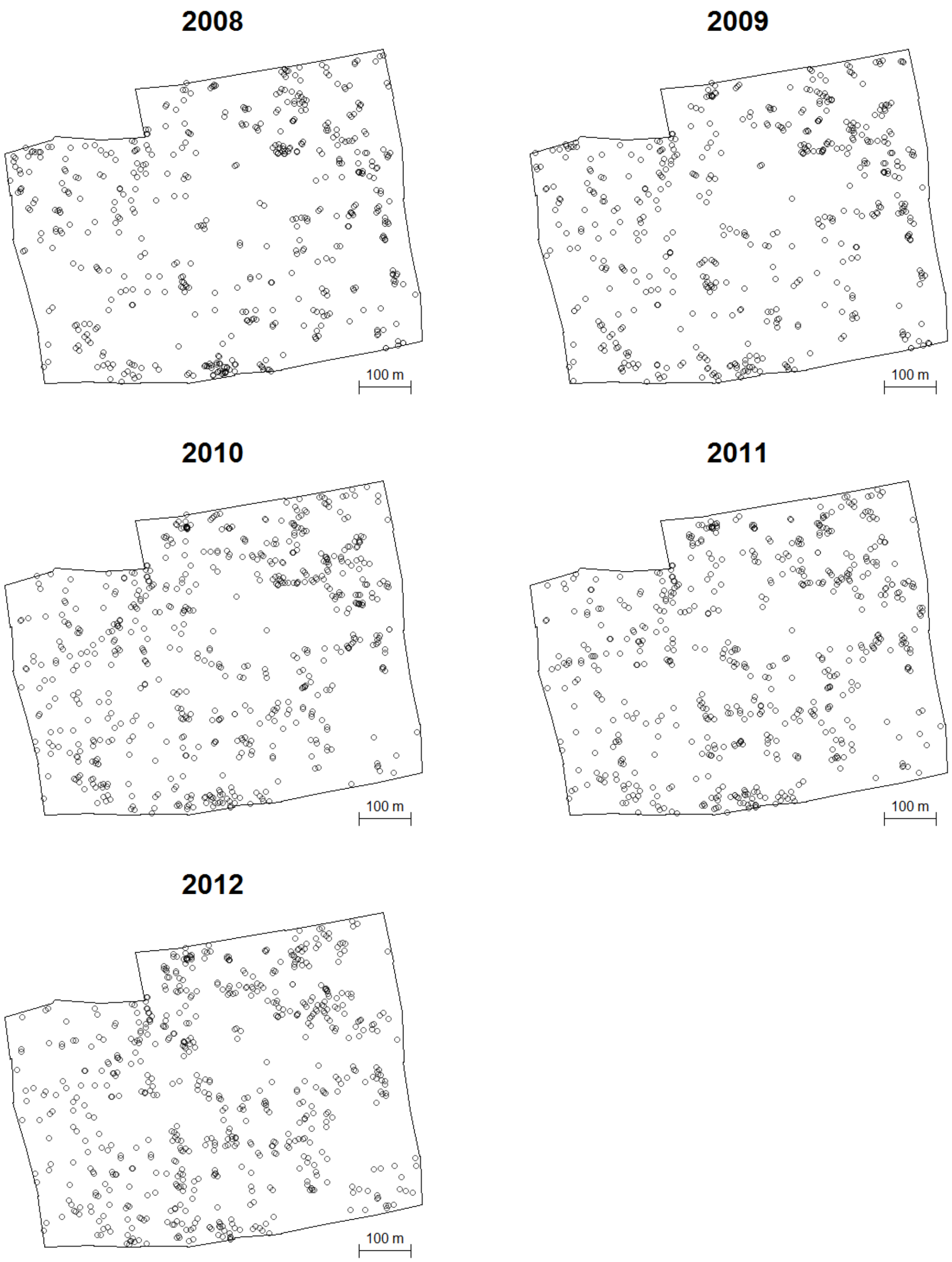
2008
100 m
2009
100 m
2010
100 m
2011
100 m
2012
100 m

Electronic supplementary material 3: Comparison of inhomogeneous cluster model fits using the *L*-statistic, from 2005 to 2012. The *L*-statistic is a variance-stabilized transformation of the Ripley's *K*, where *L(r) = sqrt(K(r)/pi)*. The dashed gray line represents the exogenous model and the gray dot-dashed line represents endogenous model. The solid black line represents the actual *L*-statistic values. Gray areas represent 95% confidence intervals, based on 999 simulations.

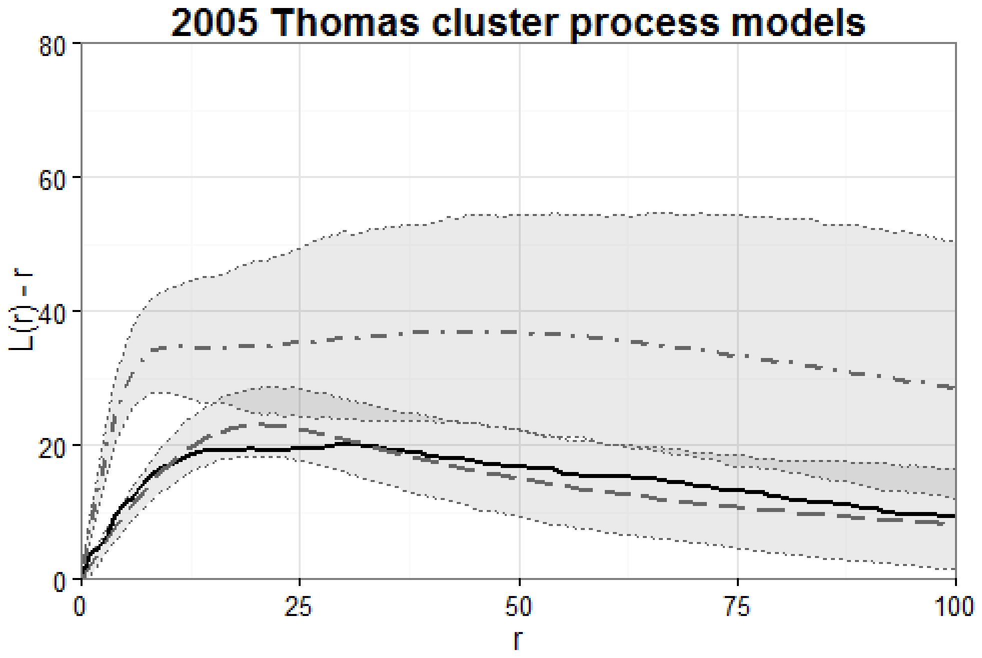

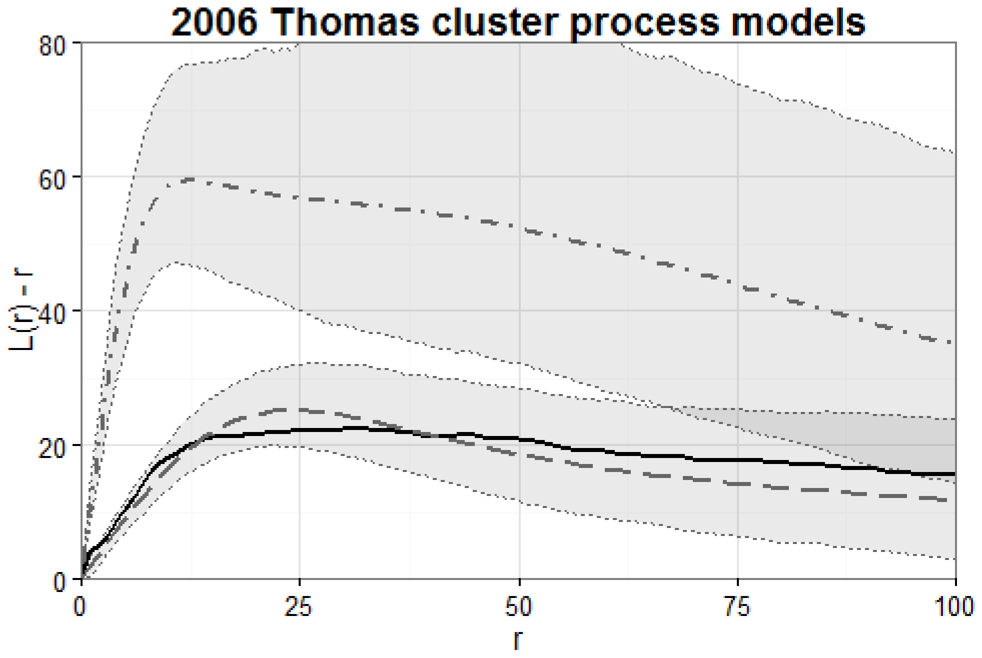

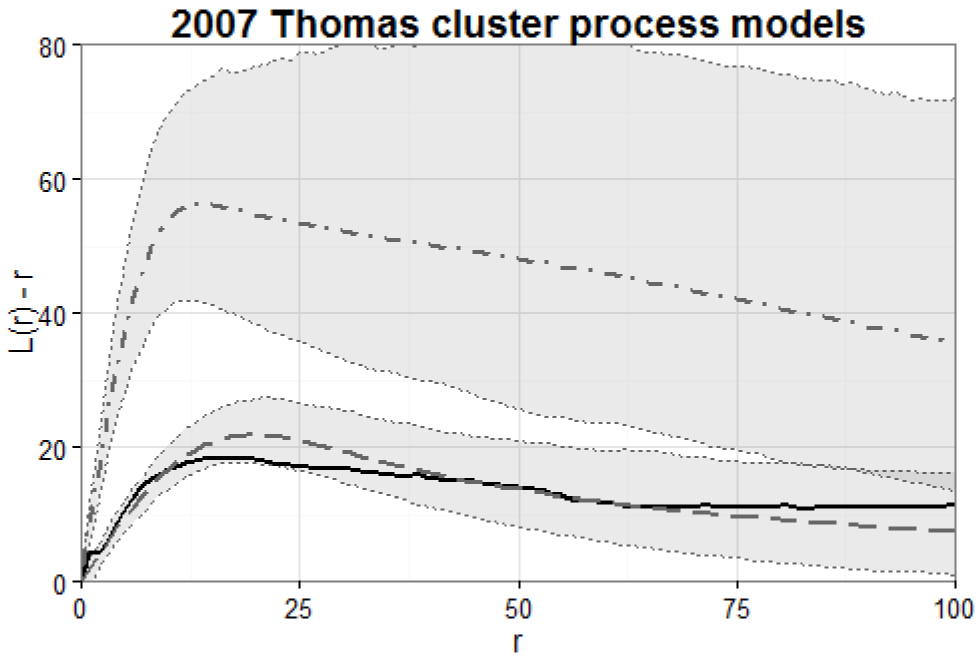
2007 Thomas cluster process models
L(r) - r
r
0
20
40
60
80
0
25
50
75
100

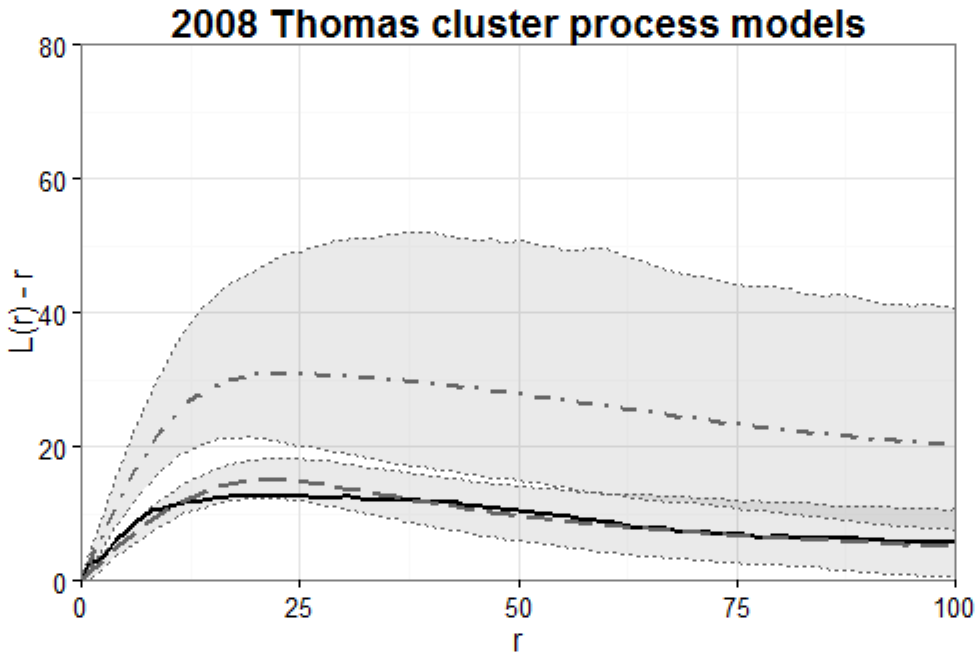
2008 Thomas cluster process models
L(r) - r
r
0
20
40
60
80
0
25
50
75
100

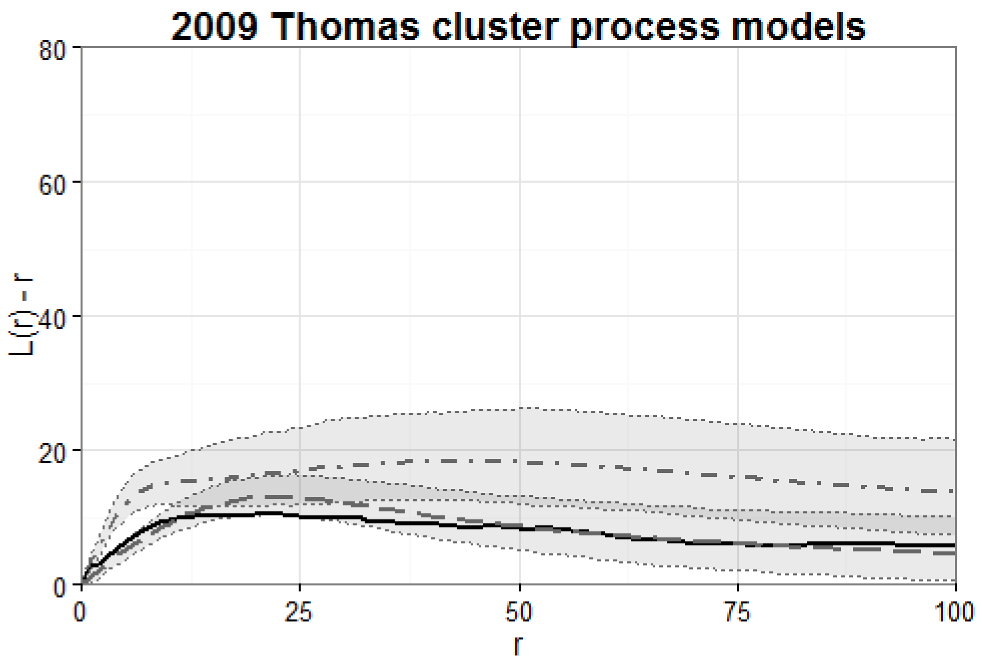
2009 Thomas cluster process models
80
60
40
20
0
L(r) - r
0
25
50
75
100
r

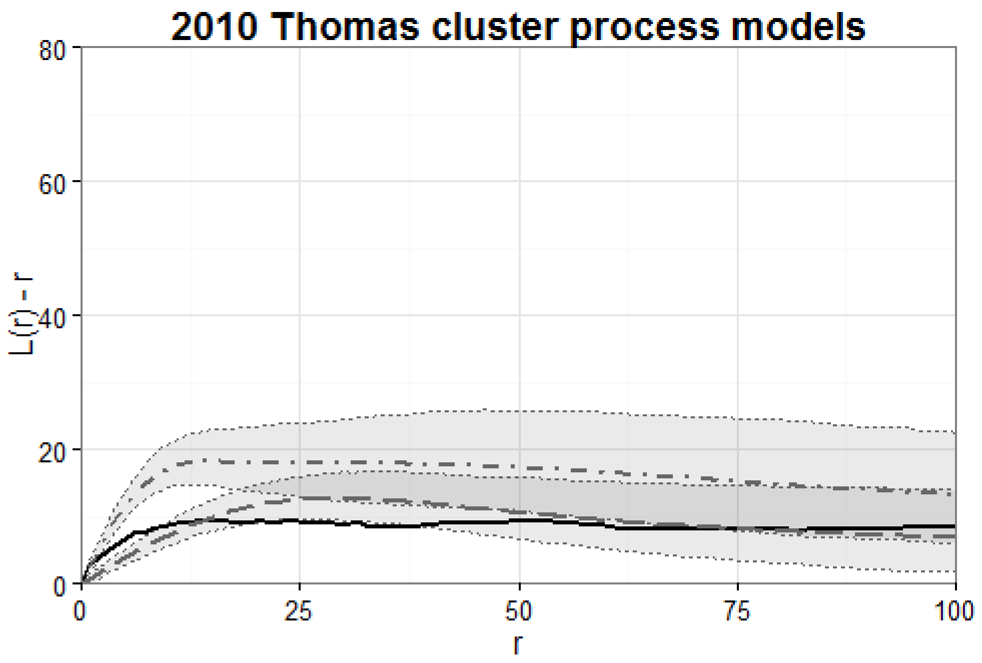
2010 Thomas cluster process models
80
60
40
20
0
L(r) - r
0
25
50
75
100
r

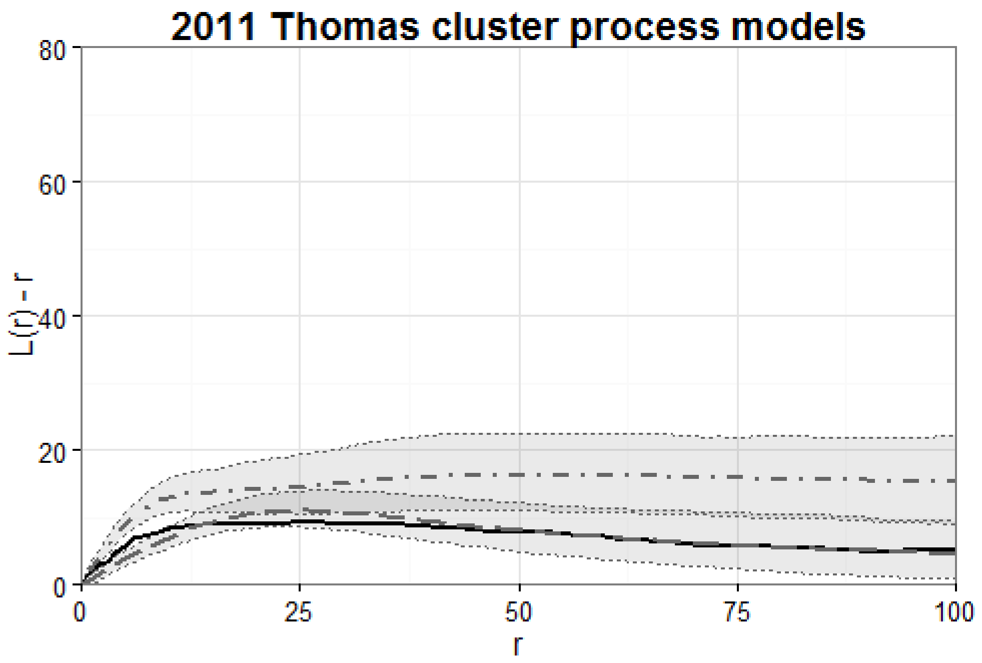

2011 Thomas cluster process models
L(r) - r
r
0
20
40
60
80
0
25
50
75
100

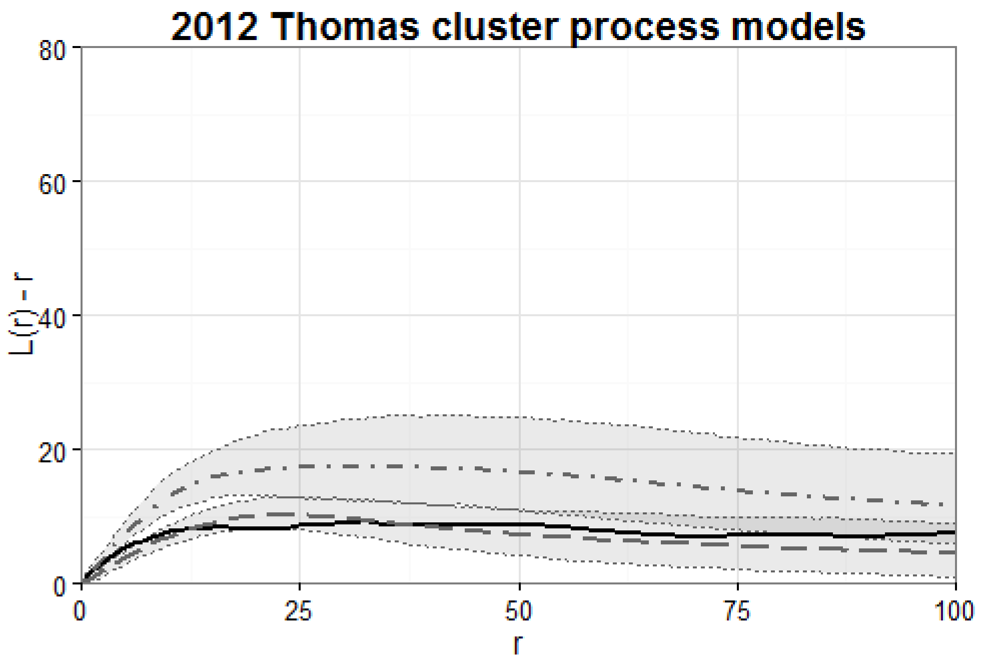

2012 Thomas cluster process models
L(r) - r
r
0
20
40
60
80
0
25
50
75
100
